# Supplementary figures and images for: The difference between cellulolytic ‘culturomes’ and microbiomes inhabiting two contrasting soil types
Source: PLoS One. 2020 Nov 20;15(11):e0242060. doi: 10.1371/journal.pone.0242060 (PMC7678988; doi:10.1371/journal.pone.0242060)

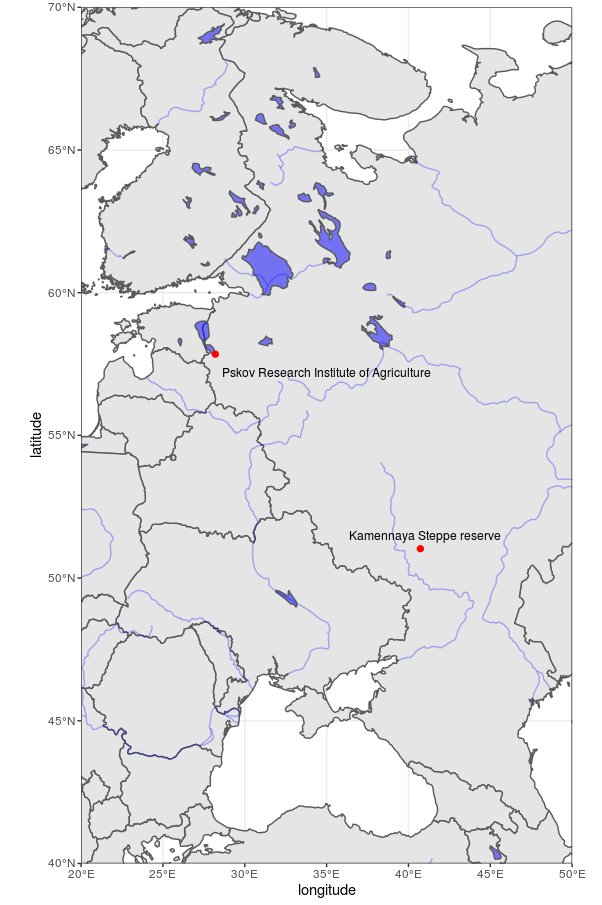

Supplement: S1 Fig — Made with Natural Earth. Free vector and raster map data @ naturalearthdata.com. (TIF) [file pone.0242060.s001.tif]
