# Supplementary material for: The difference between cellulolytic ‘culturomes’ and microbiomes inhabiting two contrasting soil types
Source: PLoS One. 2020 Nov 20;15(11):e0242060. doi: 10.1371/journal.pone.0242060 (PMC7678988; doi:10.1371/journal.pone.0242060)
Supplement: Attachment — Submitted filename: Reviewer_resp.docx [file pone.0242060.s003.docx]

Reviewer #1:

At the request of reviwer #2, the structure of the work was significantly revised. Data analysis with MiSeq was re-done, the results were rewritten, the discussion was partially rewritten.

1. In Line 34: is there a name of genus missed after Candidatus?

This part of the text was rewritten.

2. It will be more understandable, if the style to present number is uniform in the manuscript. For example, in the text, the decimal point is indicated by a period, but in the tables, comma is used to indicate decimal place.

Fixed.

3. In the section of Materials and methods, the description of sampling sites and sampling strategy is unclear. I cannot understand how many samples collected in total and whether both types of soils were sampled from both Pskov and Voronezh. Based on the results present in Fig. 2, it seems that 6 samples were taken from SP and CZ soils, respectively. However, it is unclear whether for each soil type, 3 replicates were taken from Pskov and the other three were from Voronezh, or the 6 replicates were taken from the same region. I also wonder why you said soil samples were taken from 10 different equidistant points in line 115 and in the next sentence you said that “In total, 6 replicates for each type of soil were formed”. Does it mean that each replicate in each type of soil includes 10 samples from the depth of 10 cm? It will be more understandable, if you can provide a sample list which includes the coordination of sampling sites, soil type, and sampling depth, or provide a map indicating sampling sites.

The information in the Materials and methods section was changed.

4. In the part of “Bacterial Growth on nutritional medium”, I would like to know about more detail. It is not clear liquid or solid medium used for cultivation. Additionally, it is not clear the amount of soil used to cultivate and the duration of cultivation either. Please provide more information in this part.

Explanation was added

5. In Line 175: Does the Dice methods mean “Sørensen–Dice coefficient”?

Beta and alpha diversity metrics were recalculated using UniFrac, unweighted UniFrac and Bray-Curtis metrics.

6. Please use PD whole tree and observed otus instead of the methods “PD_whole_tree” and “observed_otus” of QIIME.

Faith Index was not used in the revised version of the article.

7. Please uniform the usage of indices or indexes.

Thank you. Fixed
8. In the section of “Materials and methods”, you mentioned the indices of evenness including Faith’s index and Shannon evenness, but in “Results”, you did not show values of Faith’s index. Does PD_whole_tree means Faith’s index? If so, please uniform the name of index.

this part of the work was changed, according to the request of the second reviewer,

9. The authors did not state the methods for the ordination of Bray-curtis and Dice diversity estimates in Fig. 1.

Fixed

10. For Fig. 2, it is suggested to use percentage instead of number of seqs.

This figure have replased.

Reviewer #2:

Introduction
Line 48-49: a reason that media selection is straightforward, does it makes this a popular research subject?

This medium is often used in the institute of agricultural microbiology, where this work was done. We are looking forward to comparing the obtained metagenomic data with the previous research. This medium also known to be standard for the culturing of cellulolytic microorganisms, e.g. <https://sfamjournals.onlinelibrary.wiley.com/doi/pdf/10.1111/j.1365-2672.1997.tb03299.x>

A bit weird reasoning. Sentences 50, 51 do not really connect.
Line 58: entire biodiversity: is a bit exaggerated… ‘entire’, reword. Line 59: they used 13C-SIP?

This part of the introduction has been revised.

Line 82 and further: I appreciate the intend for ‘culturomics’, there is no need to defend this approach as being ‘old school’. It serves its purpose and NGS for other purpose so they can perfectly complement each other, depending on the research question. Line 87-88: combining culturing techniques with NGS, I agree it declines, but still I would not call it very few examples.

Fixed. Added to the discussion are examples of works comparing the cultural approach and the results of NGS.

Table 1: interpret the particle size distribution please, so sandy soil? Which soil classification type? pH in water? N, P, K total or extractable elements? Many information lacking…

P2O5 and K2O by Machigin method, N – total. pH – in water.

Line 145: picoM of primers, is little? Usually 200 nM of primers, and 200 µM of dNTPs instead of 2 nM??

5 pM is a сommonly used concentration according to Illumina MiSeq technology standards.

Line 166: which database was used for classification and which version?

Data analysis with Illumina MiSeq was redone again using a different pipeline, more correct for this work.

The taxonomic classification of phylotypes was determined using the RDP classifier based on Silva 132

Line 164: why de novo, is not recommended by Qiime, open ref-based yes.

This part of analysis has been redone

The whole analysis was done again by use of database-independent methods for out-picking
Line 174: beta-diversity in PAST3, bray Curtis? What about normalisation? What about other Unifrac based methods?

Redone. The main emphasis is on phylogenetic metrics.
Line 186: MEGA X needs a reference

This part has been changed

Line 197: wrong: the PD whole tree of culturomes cannot exceed those for bulk soils.

Thank

Table 2: in header, DP and in text, and legend SP? I cannot follow this.

This figure has been replaced

Line 198: substantial decrease, significant?

We agree. Less emphasis is placed on alpha diversity indices.

Line 200: had lower, statistics done? No..

In the corrected work, where possible, statistics are added

Because according to the data obtained below, we cannot say with certainty that the depth of sequencing for bulk soils is sufficient; comparisons of alpha diversity indices are removed from the results. In addition, the results have been significantly reworked. Where possible, statistical processing has been added.

Line 216-217: so culturomics community is similar to whole bulk soil microbiome, based on presence/absence of OTUs, species, which taxonomic level similar? Hard to believe similar in species…Next

This part has been rewritten.

line 217: they are again separate compact clusters, which statistics applied?

ANOVA-like test for beta-diversity results has been added.
Line 228: OTUs which amounts…, amounts poor English, proportions, relative abundance?

In the previous version - relative abundance.

Line 231: qualitative is usually which OTUs, quantitative: how much, RA of the OTUs

Thank

Line 235: explain this sentence again, not clear. In which both cases?

This part of analysis has been completely redone

Line 280: I don’t know how useful it is to calculate simper analyses on cultured bacteria versus total soil sample sequencing… actually this remark holds for the whole results description. Apples comparing with pears. Keep culturomics to one paragraph, and total community seq to other. Make comparisons between the total and cultured collection, ok, but not in terms of diversity please.

Direct comparisons of bulk soil and culturome were removed from the article. In addition, the discussion has been revised, comparisons have been removed based on the direct use of diversity indices.

Line 315-316: very logic cultured collection differs in diversity from total microbiome community composition, and in taxonomic composition and in core microbiome…

In the present version of the work, this part is significantly reduced

Line 318-319: very logic conclusion, no study needed for this

Thank you.

Line 320: might be the consequence, really might, it is obvious the consequence of…

Thank you. This part of discussion have been completely rewritten

Line 321: tiny portion… subset of…
Line 324-326: or did you sequence artifacts?

We cannot deny that methodological artifacts could have a significant impact on the results of the work. In a discussion in several places, an attempt was made to point this out.

Line 328-331: again all very logic. I liked the idea of culturomics, but the results description and discussion is so straightforward and disappointing. A different angle of discussion could have been followed here, what was the real purpose of the study, isolating more cellulolytic bacteria to study their function? There is no use to extensively compare beta and alpha diversity of culturomics with total microbiome sequencing, mention it in one sentence.
Rewrite some paragraphs in the discussion. Find the message you want to give to the readers.

A significant part of the analysis has been redone. Your comments have been taken into account, if possible.
