# Supplementary material for: The difference between cellulolytic ‘culturomes’ and microbiomes inhabiting two contrasting soil types
Source: PLoS One. 2020 Nov 20;15(11):e0242060. doi: 10.1371/journal.pone.0242060 (PMC7678988; doi:10.1371/journal.pone.0242060)
Supplement: Attachment — Submitted filename: Response.docx [file pone.0242060.s004.docx]

L48, L59, and L82: The way to describe the first author and the remaining coauthors should be unified.

Done

L57: phylum name is not necessary in italic form.

Done

L70: there is a space between comma and sequencing.

Done

Materials and Methods
It is suggested to use map to illustrate the sampling locations. Currently, it is not clear where the samples were taken.

Map added to supplementary.

Results
Alpha diversity of soil microbiomes and culturomes

Currently, only number of observed OTUs was used to demonstrate alpha diversity in both soil microbiomes and culturome. However, the number of observed OTUs only reflects species diversity and can not reveal the information of evenness. Therefore, it would be better to demonstrate the pattern of alpha diversity not only in number of observed OTUs, but also in other indices such as Shannon or Simpson. According to the manuscript, the term OTU is not present, but, in Figure 1A, the title of Y axis is “Number of observed OTUs”. It would be better to use phylotypes to replace OTUs.

Fixed. Added Shannon Index

Identification of the core and accessory components of soil microbiomes and culturomes
It seems that the authors compare two different types of soil and culturomes together to find out the shared phylotypes. It is suggested that the comparison should be separated based on the types of soils. SP and its culturome, and CZ and its culturome should be compared separately because they are different types of soils and originally have their specific core microbiome. The low number of shared phylotypes may be resulted from different types of
Please provide the information of total number of phylotypes in each sample.

Thank you for noticing this annoying error in the article! The paragraph was rewritten, the picture was corrected, the statistics were redone. Table with general list of core phylotypes added to supplement. Fortunately, the conclusions from this work coincide with the previous(incorrect) paragraph results.

L198: It is not necessary to put the family name in italic form.

Fixed

L198-L200: If you would like to list the family name after each genus, it would be better to add it after all genera and at the same taxonomic level, and not some in the level of family and some in the level of order.

Fixed.

L200: Pactrobactertes --> I have no idea what it is.

Typo – fixed

L203: Please modify “Verrucomicrobia phylum” to phylum Verrucomicrobia.

Done.

L204: Please modify “Candidatus Udaeobacter phylotypes” to Candidatus Udaeobacter phylotypes.

Done.

Fig. 3: It is suggested to add the stress values of NMDS analyses.

Thank you for your comment! Out of habit, I used NMDS for this dataset. Naturally, this is completely wrong - in this work the groups are very different from each other. I changed it to a more suitable PCoA.

Discussion
L269-L272: Please add citation.
In the paragraph starts from L273 to L285, similar to the pattern in the section of Introduction, the described examples seem too disperse. It would be better to focus on similar habitats.

Added. I'm afraid I could not find articles combining both approaches for the soil types studied in the article.

L289-L291: I can not fully understand the meaning of “the high degree of composition of culturome data and methodological artefacts” because the microbial composition of culturome seems simpler than bulk community. Therefore, I have no idea about the meaning of high degree of composition.

I removed the text about a higher degree of compositionality for a culturome data. For this paper, I use the more common normalization approach, without using specialized approaches for compositional data. You are right that for this article it makes no sense to get into this topic.

L289: Could you explain more about the meaning of “high resolution of the classical cultural approach”? Generally, classical cultural only can reflect a small proportion of microorganisms from field sample. Therefore, I have no idea why the classical cultural approach possesses high resolution.

Rewrote, I hope this will make the thought more accessible to the reader.

L304: Modify Xanthomonadaceae and Rhizobiaceae family to the family Xanthomonadaceae and Rhizobiaceae

Fixed

L313: Modify Paenibacillus polymyxa to P. Polymyxa

Fixed

L313: Modify Cohnella panacarvi to C. Panacarvi

Done

Thanks for your time! Your work helped us a lot.
